# Validation of the Turkish Fear of War Scale (FOWARS): Psychometric Evidence and Associations with Tolerance of Uncontrollability, Psychological Symptoms, and Personality Traits

**DOI:** 10.3390/bs16081339

**Published:** 2026-08-04

**Authors:** Feridun Kaya, Tuğba Türkkan

**Affiliations:** 1 Department of Psychology, Faculty of Letters, Atatürk University, 25240 Erzurum, Türkiye; feridun.kaya@atauni.edu.tr; 2 Department of Social Work, Faculty of Health Sciences, Gümüşhane University, 29100 Gümüşhane, Türkiye

**Keywords:** fear of war, Turkish young adults, depression, anxiety, stress, tolerance of uncontrollability, validity, reliability

## Abstract

Growing geopolitical instability has led to increasing concerns about the psychological impact of war beyond directly affected populations, highlighting the need for culturally appropriate instruments to assess fear of war. In this study, we evaluate the psychometric properties of the Turkish version of the Fear of War Scale (FOWARS) and examine its associations with sociodemographic characteristics and personality traits. A total of 643 adults across two independent samples (sample 1 = 360; sample 2 = 283) completed the FOWARS, the Tolerance of Uncontrollability Questionnaire, the Next Big Five Inventory (BFI-2), the Depression Anxiety Stress Scale-21 (DASS-21), and a sociodemographic information form. Confirmatory factor analysis supported the original two-factor structure of the FOWARS, with the Turkish version demonstrating satisfactory structural validity, convergent validity, discriminant validity, criterion-related validity, strong internal consistency, and measurement invariance across gender. Fear of war was negatively associated with tolerance of uncontrollability and positively associated with depression, anxiety, and stress. In addition, fear of war was significantly associated with gender, socioeconomic status, agreeableness, neuroticism, and conscientiousness. These findings expand the understanding of the individual determinants of fear of war, underscoring the importance of developing targeted interventions for vulnerable populations.

## 1. Introduction

Recent global crises have generated multiple stressors that have substantially affected individuals’ psychological well-being. In particular, the COVID-19 pandemic increased levels of uncertainty, fear, and anxiety across populations worldwide, resulting in widespread psychosocial distress (Ibn-Mohammed et al., 2021). Following this period, the Russia–Ukraine war, the escalation of the Israel–Palestine conflict, and increasing instability across the Middle East have brought concerns about war back to the forefront of the global agenda (Regnoli et al., 2023). Against this backdrop, the psychological impact of war now extends beyond active conflict zones, affecting not only populations directly exposed to violence but also individuals and communities in geographically neighboring or indirectly affected regions (Hajek et al., 2023).

Wars have devastating consequences and exert a profoundly negative impact on individuals’ mental health and well-being (Jalala et al., 2024), disrupting the ability of affected populations to meet basic health, economic, social, and educational needs (Spiegel et al., 2010; Tlili et al., 2024). These war-related stressors have substantial psychological repercussions, increasing the risk of depression, anxiety, post-traumatic stress disorder (PTSD), dissociation, substance use disorders, suicidal ideation, and reduced quality of life (Amsalem et al., 2025; Awad et al., 2024; Huesmann et al., 2023; Mahamid et al., 2022; Rozanov et al., 2018).

One important mechanism underlying these broader psychological effects is indirect exposure to war-related information through traditional and social media. Extensive media coverage of global crises through television and social media exposes individuals to distressing images and information from conflict zones on a daily basis, thereby increasing psychological distress and fear-related responses (Vintilă et al., 2023). Previous studies have demonstrated that repeated exposure to such negative and threatening content is associated with elevated levels of uncertainty, depression, acute stress, anxiety, and fear, even among those not directly affected by conflict (Fekih-Romdhane et al., 2024; Hajek et al., 2022). Similar patterns were observed during the COVID-19 pandemic, when excessive exposure to threat-related media content was linked to increased fear and poorer mental health outcomes (Bendau et al., 2021; Sasaki et al., 2020). Therefore, perceptions and expectations regarding the possibility and potential consequences of war may play an important role in individuals’ psychological well-being.

In this context, fear of war can be conceptualized as a multidimensional psychological construct reflecting cognitive and emotional responses to the prospect of experiencing the direct or indirect consequences of war (Najem et al., 2025). This fear is shaped by concerns about potential suffering resulting from economic instability, displacement, and emotional distress (Kalcza-Janosi et al., 2023). Global catastrophe scenarios, particularly the threat of nuclear war, may intensify fear by fostering a heightened sense of existential threat (Poikolainen et al., 2004).

In the context of unpredictable and uncontrollable life events such as war, tolerance of uncontrollability (TOU) has emerged as an important psychological construct. TOU refers to an individual’s capacity to accept and cope with situations characterized by uncontrollability (Tosun Altınöz et al., 2024). According to Barlow (2002), diminished perceived control constitutes a general psychological vulnerability that contributes to the development of a neurotic temperament and increases susceptibility to anxiety disorders. Consistent with this perspective, a lack of control over events and emotions has been associated with mood disorders as well as behavioral and physiological distress (Chorpita & Barlow, 1998; Gallagher et al., 2014; Hofmann, 2005). Recent evidence further indicates that lower levels of TOU are associated with greater psychological distress and anxiety (Akbari et al., 2024; Şener & Altan-Atalay, 2025).

Personality traits represent one of the key factors influencing how individuals perceive threats and respond to uncontrollable situations (Darvill & Johnson, 1991). Research has consistently shown that individuals high in neuroticism are more susceptible to negative affect and tend to experience greater fear and psychological distress when confronted with threatening or uncertain circumstances (Carleton et al., 2007; Dumitru et al., 2026; Tang et al., 2023). Consequently, certain personality characteristics may be associated with lower tolerance of uncontrollability and, in turn, greater fear of war. Examining these relationships may provide valuable theoretical insights into the individual differences associated with fear of war.

Beyond individual differences, cultural and geographical contexts also play an important role in shaping perceptions of war and related fears. Owing to its geopolitical position, Türkiye is located near several regions affected by ongoing or recent conflicts (including those involving Syria, Iraq, Russia and Ukraine, and Israel and Palestine). This geographical context may increase public awareness of war-related threats and susceptibility to fear of war among individuals living in Türkiye. Despite the relevance of this issue, psychometrically validated measures assessing fear of war within the Turkish cultural context remain scarce. Therefore, validating a culturally appropriate measure of fear of war for use in Türkiye is important for identifying at-risk populations and informing the development of culturally sensitive prevention and intervention strategies.

Beyond adapting the FOWARS into Turkish, our study extends previous validation research in three important ways: First, we evaluate the scale in a geopolitical context characterized by ongoing regional conflicts and heightened public awareness of war-related threats. Second, we provide the first evidence regarding the association between fear of war and tolerance of uncontrollability. Third, we examine whether scores obtained from the validated Turkish version of the FOWARS demonstrate theoretically meaningful associations with demographic characteristics and Five-Factor Model personality traits. Together, our findings provide additional evidence regarding the nomological validity of the Turkish version of the FOWARS beyond its core psychometric properties.

## 2. Current Study

The present study was conducted in two complementary phases. The first phase aimed to adapt the Fear of War Scale (FOWARS), originally developed by Kalcza-Janosi et al. (2023), to a Turkish cultural context and to examine its psychometric properties, including multiple sources of evidence related to validity and reliability. Beyond establishing the psychometric properties of an instrument, validation research should also demonstrate that the construct behaves in theoretically meaningful ways. Therefore, the second phase examined whether scores obtained from the validated Turkish version of the FOWARS were associated with selected demographic characteristics (age, gender, socioeconomic status, and marital status) and Five-Factor Model personality traits (extraversion, openness to experience, conscientiousness, agreeableness, and neuroticism). Taken together, these complementary phases provide evidence not only for the psychometric soundness of the Turkish FOWARS but also for its expected associations with relevant psychological and demographic variables.

Accordingly, the following research questions were addressed:

1. Is the FOWARS a valid instrument for assessing individuals’ levels of fear of war?
2. Is the FOWARS a reliable instrument for assessing individuals’ levels of fear of war?
3. Does the FOWARS demonstrate significant associations with criterion variables such as depression, anxiety, stress, and tolerance of uncontrollability?
4. Are demographic variables (age, gender, socioeconomic status, and marital status) significantly associated with fear of war?
5. Are Five-Factor Model personality traits significantly associated with fear of war?

## 3. Method

### 3.1. Participants

Two independent samples were recruited using a convenience sampling strategy based on voluntary participation. The first sample comprised 360 participants, with a mean age of 21.37 years (SD = 2.57; range = 18–35), while the second sample comprised 283 participants, with a mean age of 21.40 years (SD = 3.27; range = 18–45). Examination of the demographic characteristics indicated that the majority of participants in both samples were female, single, and of middle socioeconomic status. Although the age ranges extended into later adulthood, the relatively low mean ages indicate that both samples were predominantly composed of young adults. Accordingly, the samples should not be considered representative of the broader adult population. Detailed participant characteristics are presented in Table 1. Factor analytic guidelines suggest a minimum of approximately 15 participants per observed variable (Stevens, 2009). In the second phase, an a priori power analysis was conducted to determine the required sample size using G*Power 3.1 software (Faul et al., 2009). For the regression analyses, a model including nine predictor variables was specified, with an alpha level of 0.05, statistical power (1 − *β*) of 0.95, and a medium effect size (*f*^2^ = 0.15). Based on these parameters, the minimum required sample size was calculated as 166 participants (Balkin & Sheperis, 2011). To enhance the robustness and generalizability of the findings, data were collected from 283 participants, thereby exceeding the minimum required sample size and ensuring adequate statistical power.

### 3.2. Procedure

Several steps were followed to translate the FOWARS into Turkish. First, the original items were independently translated into Turkish by seven doctoral-level academics in psychology who were proficient in English and who did not belong to the research team. In the second stage, the research team reviewed, compared, and synthesized the translated versions through an expert consensus process by evaluating their semantic, conceptual, and cultural equivalence. No substantive semantic or conceptual discrepancies were identified, and a consensus Turkish version was established. In the third stage, the consensus Turkish version was independently back-translated into English by two bilingual experts who did not belong to the research team and who had not participated in the forward translation process. The back-translated version was compared with the original scale to evaluate semantic and conceptual equivalence, and no substantial discrepancies requiring item revision were identified. In the fourth stage, the original English items and the back-translated version were administered to 52 students of English Language and Literature (40 females, 12 males) who were proficient in both Turkish and English, with an interval of a few days between administrations (the Turkish version was administered first, followed by the English version). A strong positive correlation was observed between the two administrations (r = 0.86, *p* < 0.001), providing supportive evidence for linguistic equivalence. This evidence was not considered sufficient on its own but was rather interpreted alongside the independent forward translation, the expert evaluation of semantic, conceptual, and cultural equivalence, and the independent back-translation process as one component of the overall cultural adaptation procedure before proceeding to the psychometric evaluation of the Turkish version.

In the first data collection phase, an online survey including a demographic information form, the FOWARS, the Depression Anxiety Stress Scale, and the Tolerance of Uncontrollability Questionnaire was distributed via social media platforms. Participants who provided informed consent by checking the relevant box were included in the study, and completion time was approximately 15 min. Data for this phase were collected between 17 April and 25 April 2026. In the second phase, the aim was to examine the associations of demographic variables and Five-Factor Model personality traits with fear of war. Accordingly, an online form including a demographic information form, the Five-Factor Personality Scale, and the FOWARS was administered. Completion time for this phase was approximately 10 min. Data were collected between 1 May and 7 May 2026. Our study was approved by the Atatürk University Social Sciences Ethics Committee (Decision No: 8/115, dated 14 April 2026; Document No: E.88656144-000-2600129515).

### 3.3. Instruments

#### 3.3.1. The Fear of War Scale (FOWARS)

The FOWARS, developed by Kalcza-Janosi et al. (2023), was designed to assess individuals’ levels of fear of war. The scale consists of 13 items across two subdimensions: an experiential subscale (seven items) and a physiological subscale (six items). Responses are rated on a 5-point Likert scale ranging from 1 (“strongly disagree”) to 5 (“strongly agree”), with higher scores indicating greater fear of war. In the original validation study, analyses conducted on two independent samples (*N* = 1131) demonstrated satisfactory exploratory factor analysis (EFA) and confirmatory factor analysis (CFA) results (χ^2^ = 303.490, df = 64, *p* < 0.001, χ^2^/df = 4.742, CFI = 0.938, GFI = 0.910, TLI = 0.924, NFI = 0.946, SRMR = 0.044). Reliability analysis indicated that Cronbach’s alpha coefficients for the experiential subscale were 0.88 in the first sample and 0.92 in the second sample, while the physiological subscale yielded an alpha coefficient of 0.88 in both samples. Cronbach’s alpha values for the overall scale were 0.91 and 0.93 for the first and second samples, respectively. Detailed findings regarding the validity and reliability of the measurement instrument are presented in Section 4.

#### 3.3.2. The Tolerance of Uncontrollability Questionnaire (TOUQ)

The TOUQ, developed by Hay et al. (2022), was designed to assess adults’ TOU in life situations. Adaptation of the scale into Turkish was conducted by Tosun Altınöz et al. (2024). The instrument consists of 19 items rated on a 7-point Likert scale ranging from 1 (“strongly disagree”) to 7 (“strongly agree”), with higher scores indicating greater tolerance of uncontrollability. Regarding its reliability properties, the original scale demonstrated excellent internal consistency, with a Cronbach’s alpha coefficient of 0.97 (Hay et al., 2022). In the Turkish adaptation study, the Cronbach’s alpha value was reported as 0.93 (Tosun Altınöz et al., 2024). In the present study, the scale also showed excellent reliability, with a Cronbach’s alpha coefficient of 0.96.

#### 3.3.3. The Depression Anxiety Stress Scale-21 (DASS-21)

The DASS-21 is a shortened version of the original 42-item scale developed to assess individuals’ levels of depression, anxiety, and stress (Lovibond & Lovibond, 1995). The instrument comprises 21 items in total, with seven items allocated to each of the three subscales. Responses are rated on a 4-point Likert scale ranging from 0 (“never”) to 3 (“almost always”). Cronbach’s alpha coefficients were determined to be 0.94 for depression, 0.87 for anxiety, and 0.91 for stress (Antony et al., 1998). In the Turkish adaptation study conducted by Sariçam (2018), Cronbach’s alpha coefficients were reported as 0.85 for depression, 0.80 for anxiety, and 0.77 for stress. In the present study, the scale demonstrated Cronbach’s alpha coefficients of 0.92 for depression, 0.93 for anxiety, and 0.90 for stress, and a total scale reliability of 0.96.

#### 3.3.4. The Next Big Five Inventory (BFI-2)

The BFI-2, developed by Soto and John (2017a, 2017b), was designed to assess the five major dimensions of personality: open-mindedness, conscientiousness, extraversion, agreeableness, and neuroticism. The full version of the scale consists of 60 items and is rated on a 5-point Likert scale ranging from 1 (“strongly disagree”) to 5 (“strongly agree”). Previous psychometric evaluations have demonstrated Cronbach’s alpha coefficients of 0.83 for extraversion, 0.79 for agreeableness, 0.86 for conscientiousness, 0.89 for neuroticism, and 0.81 for open-mindedness (Soto & John, 2017b; Soto, 2021). In addition to the full form, the BFI-2 has two abbreviated versions: a 30-item short form (BFI-2-S) and a 15-item very short form (BFI-2-XS) (Cemalcilar et al., 2021). Adaptation of the BFI-2 into Turkish was conducted by Cemalcilar et al. (2021), who reported reliability coefficients separately for student and community samples. In the student sample, Cronbach’s alpha values were 0.82 for open-mindedness, 0.87 for conscientiousness, 0.87 for extraversion, 0.79 for agreeableness, and 0.87 for neuroticism, while, in the community sample, the corresponding values were 0.64, 0.75, 0.68, 0.78, and 0.63, respectively. In the present study, the 15-item very short form (BFI-2-XS) was used. Cronbach’s alpha coefficients in the current sample were 0.75 for open-mindedness, 0.78 for conscientiousness, 0.74 for extraversion, 0.72 for agreeableness, and 0.71 for neuroticism.

### 3.4. Data Analysis

Confirmatory factor analyses (CFAs) were performed to evaluate the structural validity of the Turkish version of the FOWARS. This technique is appropriate for testing the fit of theoretically established measurement models (Tabachnick & Fidell, 2014). CFAs were conducted in JASP 0.16.3 using the Diagonally Weighted Least Squares (DWLS) estimator, which is widely recommended in the literature for the analysis of ordinal data (DiStefano & Morgan, 2014; Li, 2016a, 2016b, 2021). Various fit indices were used to evaluate the model fit. For these indices, >0.90 for CFI and NFI and <0.08 for RMSEA and SRMR were considered acceptable fit criteria (Hu & Bentler, 1999; Kline, 2016). An RMSEA < 0.05 indicates good fit, between 0.05 and 0.08 indicates acceptable fit, and above 0.10 indicates poor fit (Brown, 2015). In addition to model fit, analyses were conducted considering the need for the scale’s factor loadings to be above the 0.32 cut-off point emphasized in the literature (Tabachnick & Fidell, 2014). The reliability of the scale was evaluated by calculating the internal consistency coefficient, with values of 0.70 or higher generally considered indicative of acceptable internal consistency (Hair et al., 2019). Since Cronbach’s alpha coefficient is more robust to most assumption violations, it is suggested that McDonald’s omega (ω) coefficient be calculated as a more stable indicator of internal consistency in psychometric research (McNeish, 2018). In addition, convergent validity (based on factor loadings and AVE values), discriminant validity (using the HTMT and Fornell–Larcker criteria), and composite reliability were assessed in the study. AVE values are expected to be above 0.50, HTMT values below 0.85, and composite reliability values above 0.70 (Fornell & Larcker, 1981; Henseler et al., 2015; Yusefi et al., 2025).

In this study, measurement invariance across gender groups was tested in accordance with the steps recommended by Bowen and Masa (2015). Because the data were ordinal, multi-group confirmatory factor analysis (MG-CFA) was conducted using the DWLS estimator. In comparisons of nested models, changes in CFI and RMSEA were considered instead of the χ^2^ statistic. Values of ΔCFI of 0.01 or less and ΔRMSEA below 0.015 were interpreted as evidence that measurement invariance had been established (Byrne, 2010; Chen, 2007; Cheung & Rensvold, 2002). Measurement invariance was examined sequentially at the configural, metric, and scalar levels. Configural invariance was tested to determine whether the same factor structure was present across gender groups. Metric invariance was subsequently examined by constraining the factor loadings to equality across groups. Finally, scalar invariance was tested by constraining both the factor loadings and item thresholds to equality, thereby determining whether latent mean scores could be meaningfully compared across gender groups (Chen, 2007; Putnick & Bornstein, 2016; Watters et al., 2019). Following the evaluation of the construct validity of the FOWARS, Pearson correlation analysis was conducted to examine the relationships among fear of war, depression, anxiety, stress, and tolerance of uncontrollability, as all variables demonstrated a normal distribution.

Categorical predictors were coded prior to the regression analysis. Gender was coded as 0 = male and 1 = female, with males serving as the reference category. Marital status was coded as 0 = single and 1 = married, with single participants serving as the reference category. Socioeconomic status comprised three categories—low, middle, and high—with low socioeconomic status as the reference category. Hierarchical regression analysis was performed to examine the associations of selected sociodemographic and personality characteristics with fear of war. In the analysis, the predictor variables were entered into the model in successive stages (blocks), with each block representing a separate regression model. At each stage of the analysis, various regression statistics were calculated, most notably the coefficient of determination (*R*^2^). One of the key indicators in hierarchical regression analysis is the change in *R*^2^ (Δ*R*^2^), which represents the additional contribution of newly entered predictors to the variance in the dependent variable after controlling for the effects of the variables included in the preceding stages (Field, 2013). To test for autocorrelation, the Durbin–Watson statistic was calculated (D–W = 1.30), and the assumption was considered met, as the value fell within the acceptable range of 1 to 3 (Durbin & Watson, 1950). Multicollinearity was assessed using VIF and tolerance values, with recommended VIF values < 5 and tolerance values > 0.20 (Hair et al., 2011, 2019). In the present analysis, VIF values ranged from 1.22 to 1.33, and tolerance values ranged from 0.76 to 0.82, indicating no multicollinearity. Cook’s D values were calculated to identify multivariate outliers, and all values were below 1 (Cook, 1977; Cook & Weisberg, 1982), indicating that all assumptions were met. All analyses were conducted using IBM SPSS Statistics 21.0.

## 4. Results

### 4.1. Validity and Reliability of the Turkish Form of the FOWARS

#### 4.1.1. Structural Validity

**Figure 1:**
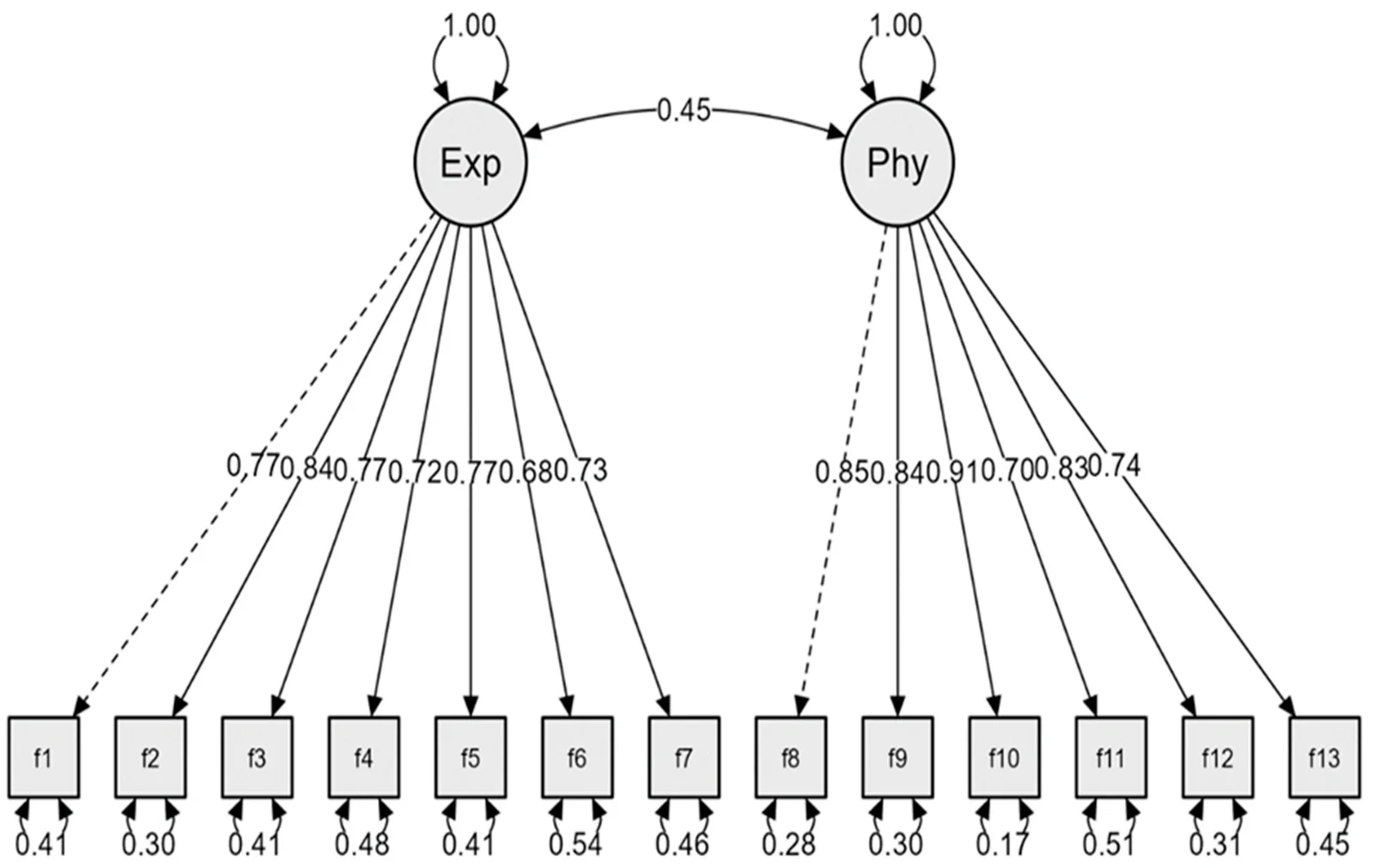
Two-factor CFA results for the FOWARS. **Note.** Dashed lines indicate fixed parameters, whereas solid lines indicate freely estimated factor loadings.

A two-factor CFA model was tested for the FOWARS. Our results indicated that factor loadings for the experiential subscale ranged from 0.68 to 0.84, while those for the physiological subscale ranged from 0.70 to 0.91. Details of the CFA model are presented in Figure 1.

The factor loadings of the scale were considerably higher than the cut-off values reported in the literature. Model fit indices indicated a good to excellent fit (χ^2^ = 115.613, df = 64, χ^2^/df = 1.806, NFI = 0.987, CFI = 0.989, RMSEA = 0.047 [90% CI = 0.033–0.061], and SRMR = 0.065). Overall, these results support the adequacy of the model and confirm the two-factor structure of the FOWARS in the Turkish sample. Convergent validity, composite reliability, and discriminant validity were also examined. The average variance extracted (AVE) values were 0.572 for the experiential factor and 0.666 for the physiological factor, exceeding the recommended threshold of 0.50. Composite reliability (CR) values were 0.90 and 0.92 for the experiential and physiological factors, respectively. The correlation between the two factors was 0.45, corresponding to a squared inter-factor correlation of 0.203. Because the AVE value of each factor exceeded the squared inter-factor correlation, the Fornell–Larcker criterion was met. In addition, the HTMT value was 0.60, which was below the recommended cut-off values of 0.85 and 0.90. The square root of the average variance extracted was 0.756 for the experiential factor and 0.816 for the physiological factor. Both values exceeded the correlation between the two latent factors (r = 0.45), indicating that each construct shared more variance with its own indicators than with the other construct. These findings provide additional evidence supporting the convergent validity, reliability, and discriminant validity of the two-factor measurement model. Standardized factor loadings for the two-factor CFA model are presented in Table 2.

#### 4.1.2. Criterion-Related Validity

In the criterion-related validity analysis, correlations were examined between scores of the FOWARS and the criterion measures of DASS-21 and the TOUQ based on a sample of 360 participants. The data from the applied scales were normally distributed, and the correlation coefficients, along with skewness and kurtosis values, are presented in Table 3.

Examination of the correlation coefficients revealed a statistically significant negative association between fear of war and TOU (r = −0.21, *p* < 0.001). In contrast, fear of war was positively associated with depression (r = 0.21, *p* < 0.001), anxiety (r = 0.29, *p* < 0.001), and stress (r = 0.24, *p* < 0.001). These findings provide evidence for the criterion-related validity of the FOWARS.

#### 4.1.3. Measurement Invariance Results

Measurement invariance across gender groups was tested sequentially at the configural, metric, and scalar levels. The configural model demonstrated an acceptable fit to the data, with χ^2^(128) = 282.520, χ^2^/df = 2.207, CFI = 0.948, SRMR = 0.075, and RMSEA = 0.082. The metric invariance model also showed an acceptable fit, with χ^2^(139) = 298.734, χ^2^/df = 2.149, CFI = 0.942, SRMR = 0.081, and RMSEA = 0.080. Compared with the configural model, the changes were ΔCFI = 0.006 and ΔRMSEA = 0.002. As both values were within the recommended thresholds, metric invariance was supported. The scalar invariance model yielded χ^2^(150) = 355.143, χ^2^/df = 2.368, CFI = 0.934, SRMR = 0.079, and RMSEA = 0.087. Compared with the metric model, the changes were ΔCFI = 0.008 and ΔRMSEA = 0.007. Because both values remained below the recommended cut-off values, scalar invariance was also supported. This finding indicates that latent mean scores can be meaningfully compared across gender groups (Table 4).

#### 4.1.4. Reliability Results

The reliability of the FOWARS was assessed using Cronbach’s alpha and McDonald’s omega coefficients based on the data from 360 participants. The experiential subscale demonstrated high reliability (α = 0.897, ω = 0.908), as did the physiological subscale (α = 0.941, ω = 0.937). For the overall scale, the results were α = 0.930 and ω = 0.949, respectively, indicating that both the subscales and the total scale possess strong internal consistency.

### 4.2. Associations of Sociodemographic Characteristics and Five-Factor Personality Traits with Fear of War

Hierarchical multiple linear regression analysis was conducted to examine the associations between age, gender, marital status, socioeconomic status, the Five-Factor personality traits, and fear of war. The results of the analysis are presented in Table 5.

In the first step of the hierarchical regression analysis, age, gender, marital status, and socioeconomic status were entered into the model. These variables explained 15% of the variance in fear of war scores (*F*_(4,278)_ = 11.859, *p* < 0.001). Among the sociodemographic variables, gender (*β* = 0.24, *p* < 0.001) and socioeconomic status (*β* = 0.21, *p* < 0.001) were significantly associated with fear of war. Specifically, female participants reported higher levels of fear of war, and fear of war increased with higher socioeconomic status.

**Figure 2:**
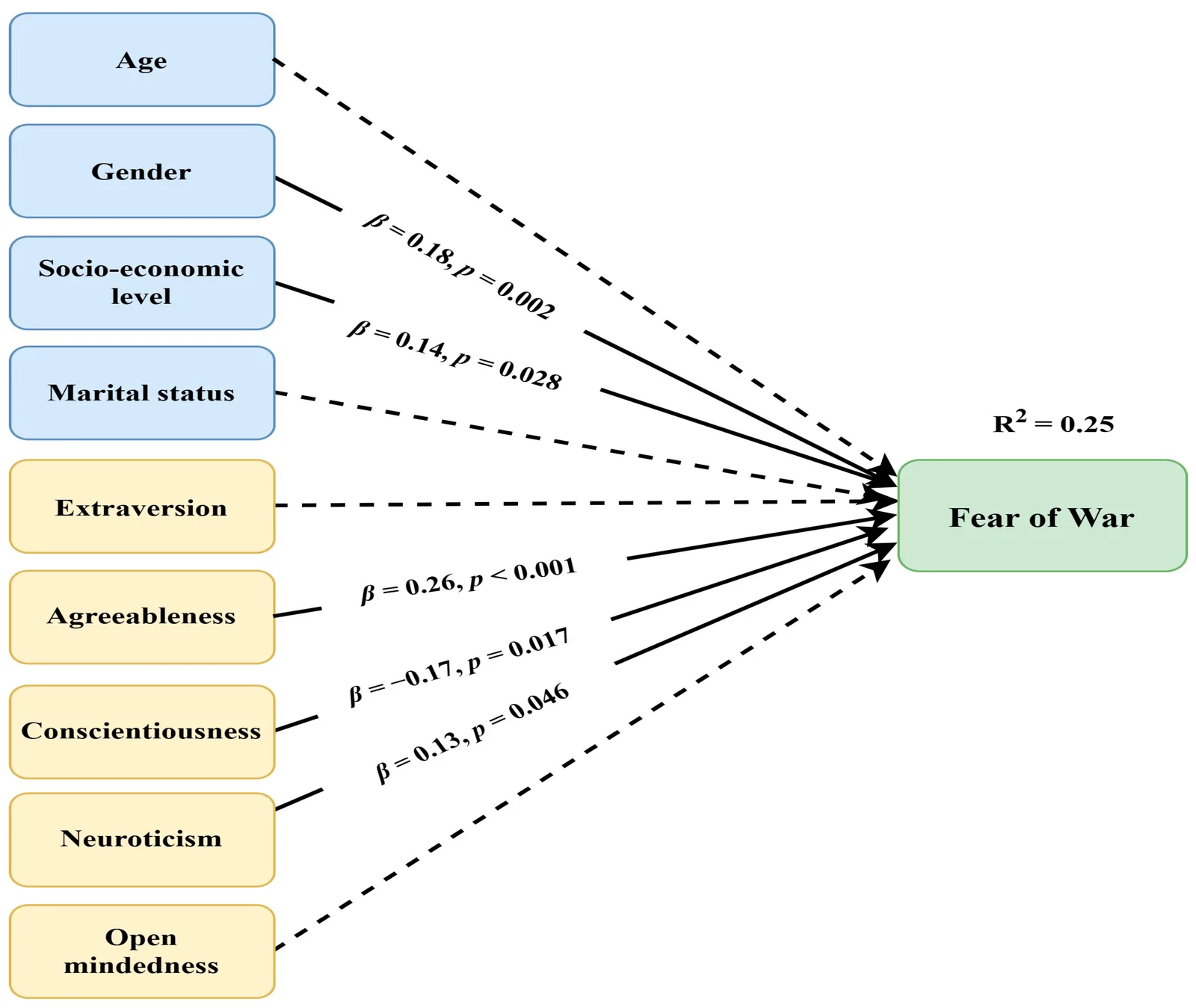
Variables significantly associated with fear of war. **Note.** Dashed lines indicate non-significant coefficients, whereas solid lines indicate statistically significant coefficients.

In the second step, the five personality dimensions were added to the model (see Model 2), resulting in an additional 10% increase in explained variance (Δ*R*^2^ = 0.100, Δ*F*_(5,273)_ = 7.211, *p* < 0.001). The results indicated that agreeableness (*β* = 0.26, *p* < 0.001) and neuroticism (*β* = 0.13, *p* = 0.046) were positively associated with fear of war, whereas conscientiousness was negatively associated with fear of war (*β* = −0.17, *p* = 0.017). Extraversion and open-mindedness were not significantly associated with fear of war. Overall, these findings indicate that personality traits were significantly associated with fear of war beyond the sociodemographic characteristics included in the model. As a result, the model explained approximately 25% of the variance in fear of war. Although this represents a meaningful contribution, the remaining variance may be attributable to psychological, social, demographic, and contextual factors not examined in the present study. The results are illustrated in Figure 2.

## 5. Discussion

In the present study, we initially focused on the adaptation and validation of the FOWARS for use in a Turkish context. Our findings demonstrated that the Turkish version of the scale possesses satisfactory psychometric properties. CFA supported the original two-factor structure, with all items loading significantly on their respective factors and the overall model showing excellent fit. The two dimensions were retained as experiential fear and physiological fear: experiential fear reflects individuals’ subjective concerns, worries, and emotional reactions related to war, whereas physiological fear refers to bodily responses triggered by perceived war threats, such as increased heart rate, sweating, or trembling (Kalcza-Janosi et al., 2023). Our findings also provided support for the convergent and discriminant validity of the two-factor structure. The AVE values for both the experiential and physiological factors exceeded the recommended threshold of 0.50, indicating that each latent construct explained a sufficient proportion of variance in its respective indicators. In addition, the high composite reliability coefficients, together with Cronbach’s alpha and McDonald’s omega coefficients, supported the strong internal consistency of both dimensions and the total scale. Evidence for discriminant validity was obtained from multiple complementary criteria. Specifically, the AVE values for both factors exceeded the squared inter-factor correlation, the square root of the AVE for each factor was greater than the correlation between the two latent constructs, and the HTMT value was below the recommended cut-off values. Taken together, these findings suggest that the experiential and physiological factors are empirically distinguishable yet moderately related dimensions of the same broader construct, capturing conceptually related but non-redundant aspects of fear of war.

The scale scores showed modest correlations with depression, anxiety, stress, and tolerance of uncontrollability, all in the theoretically expected directions. These findings provide supportive evidence for criterion-related validity while also indicating that the measured constructs are related but conceptually distinct. Consistent with previous research, greater fear of war has been associated with higher levels of depression and anxiety (Hajek et al., 2023), lower well-being, and greater future-oriented concerns (Najem et al., 2025). Similarly, a large-scale study conducted during the early stages of the Russia–Ukraine war reported elevated anxiety levels across all age groups, in some cases exceeding those observed during the strictest phases of the COVID-19 pandemic (Gottschick et al., 2023). Collectively, these findings support the view that fear of war is associated with psychological distress even among populations that are not directly exposed to armed conflict.

An additional strength of the present study is that the Turkish version of the FOWARS demonstrated configural, metric, and scalar measurement invariance across gender. These findings indicate that the scale measures the same underlying construct in women and men and that observed gender differences are unlikely to reflect measurement bias. Establishing measurement invariance is an important component of contemporary psychometric validation because it provides evidence that comparisons across groups are meaningful and methodologically sound. Accordingly, these findings strengthen the psychometric evidence for the Turkish version of the FOWARS and provide a sound methodological basis for future studies comparing fear of war across gender groups. Taken together, our findings indicate that the Turkish version of the FOWARS is a psychometrically sound instrument for assessing fear of war and can be used for meaningful comparisons across gender groups.

Previous research has consistently shown that war is associated with a wide range of adverse psychological consequences, including depression, anxiety, post-traumatic stress symptoms, reduced well-being, and disruptions to daily life (Alhamood et al., 2026; Hoffmann et al., 2024; Lim et al., 2022; Pettersson et al., 2021). Importantly, these effects are not limited to directly exposed populations but may also extend to individuals living far from conflict zones through repeated exposure to war-related information and media coverage (Baigozhina et al., 2020; Chudzicka-Czupała et al., 2023; Malecki et al., 2023). Consistent with this literature, the criterion-related validity findings of the present study further support the association between fear of war and psychological distress among individuals who are geographically distant from armed conflicts. Although media exposure was not examined in the present study, its potential role in shaping fear of war warrants further investigation.

The findings of the present study indicated that fear of war is negatively associated with TOU. To our knowledge, this relationship has not been examined in previous studies on fear of war. Controllability refers to both actual and perceived control over external stressors and internal experiences (Hancock & Bryant, 2018). Cognitive models propose that beliefs related to lack of control and catastrophic interpretations contribute to psychological distress by fostering a sense of helplessness in managing emotional reactions (Ehlers & Clark, 2000). This process may activate negative beliefs following experiences that challenge perceptions of controllability, which in turn can lead to difficulties in emotion regulation, excessive reliance on avoidance-based coping strategies, and increased emotional distress (Ehlers & Clark, 2000). The present findings suggest that fear of war is linked not only to perceptions of external threat but also to how individuals perceive and manage uncertainty. Individuals with lower TOU may be more likely to interpret uncertain and uncontrollable situations, such as the possibility of war, as threatening, resulting in heightened vigilance and emotional distress even in the absence of an immediate threat. In contrast, individuals with greater TOU may evaluate uncontrollability in a more balanced and contextual manner. Consistent with this interpretation, previous research has shown that beliefs related to lack of control are associated with increased emotional distress and stronger stress responses (Hancock & Bryant, 2020). Accordingly, the present findings suggest that lower TOU may be associated with greater fear of war through differences in how uncertain and uncontrollable situations are appraised.

Beyond our psychometric findings, we also examined whether selected demographic characteristics and personality traits were associated with fear of war. Our findings indicated that age and marital status were not significantly associated with fear of war, whereas women reported higher levels of fear than men, and individuals with higher socioeconomic status reported greater fear than those with lower socioeconomic status. Existing research on this topic remains limited and has often approached fear from different perspectives. For example, an ethnographic study conducted during the Nepal conflict found that fear levels varied according to personality characteristics, life experiences, coping abilities, and available social resources (Pettigrew & Adhikari, 2017). In addition, research on crime-related fears may provide useful insights into understanding fear of war. Studies in this area have shown that fear is associated with perceived vulnerability, previous victimization, gender, age, and socioeconomic conditions (Cossman et al., 2016; Lane & Meeker, 2004; Rader et al., 2012; Schafer et al., 2006; Snedker, 2012).

Consistent with the findings of the present study, previous research has shown that women tend to report higher levels of fear than men during periods of war (Hale, 1996; Williams et al., 2018). One possible explanation for this finding is that gender-role socialization may influence how individuals perceive and respond to potential threats. Research in social psychology suggests that men and women differ in their appraisal of risk and victimization (Das-Gupta & Li, 1999). Women have been reported to perceive themselves as more vulnerable, report lower perceived coping resources, and exhibit greater sensitivity to potential threats (Das-Gupta & Li, 1999; Sutton & Farrall, 2005). They may also be more attentive to danger-related cues and more likely to perceive uncertain situations as risky (Kimhi & Shamai, 2006). Conversely, traditional masculine norms may discourage men from expressing fear because such emotions may be viewed as inconsistent with socially prescribed gender roles (Gilchrist et al., 1998). Taken together, these findings suggest that gender differences in fear may be associated, at least in part, with gender-role socialization and societal expectations.

Findings regarding the relationship between age and fear have been inconsistent across studies. Whereas Cohen-Shani et al. (2026) reported higher levels of fear of war among younger adults following the Israel–Hamas War, Hajek et al. (2023) found no significant association between age and fear of war in a general adult population, consistent with the present findings. Similar inconsistencies have also been reported in the fear-of-crime literature. Although some studies have suggested that older adults experience greater fear (Pantazis, 2000; Cossman & Rader, 2011), more recent evidence indicates that this association becomes non-significant after accounting for factors such as health status, gender, education, and neighborhood characteristics (Hanslmaier et al., 2018; Ralston et al., 2025). Together, these findings suggest that individual and contextual characteristics may be more closely associated with fear than chronological age alone.

Nevertheless, this finding should be interpreted with caution because both samples consisted predominantly of young adults and exhibited a relatively restricted age range. This limited age variability may have reduced the likelihood of detecting age-related differences in fear of war. Accordingly, the present findings should not be interpreted as evidence that age is unrelated to fear of war across the broader population. Future research should therefore examine more age-diverse and representative samples to determine whether the association between age and fear of war differs across the lifespan.

In the present study, we found no significant association between marital status and fear of war. Previous studies, although limited in number, have reported higher levels of fear of war or greater psychological distress among married individuals than among single individuals (Hajek et al., 2023; Kurapov et al., 2022). One possible explanation is that marital status may be associated with additional family responsibilities and concerns regarding the safety and well-being of loved ones (Williams et al., 2018). However, the present findings suggest that fear of war may represent a broader response to perceived societal threat that is not necessarily dependent on marital status. Nevertheless, this finding should be interpreted with caution because the sample consisted predominantly of young and unmarried adults. Future studies including more demographically diverse samples are needed to clarify whether marital status is associated with fear of war across different populations.

In the present study, we found that participants with higher socioeconomic status reported greater fear of war than those with lower socioeconomic status. Because previous studies directly examining the relationship between socioeconomic status and fear of war are lacking, this finding should be interpreted cautiously. Although research on fear of crime has generally reported higher levels of fear among individuals with lower socioeconomic status (McKee & Milner, 2000), war may represent a qualitatively different type of threat that extends beyond concerns about personal safety. One possible explanation is that individuals with greater economic resources may perceive themselves as having more to lose during periods of armed conflict. Alternatively, greater access to news media and international sources of information may increase awareness of war-related threats among individuals with higher socioeconomic status. However, these explanations remain speculative because our study did not directly examine the factors that might account for this association. Future research should therefore investigate this association by examining factors such as perceived threat, economic concerns, and exposure to war-related information.

Regression analyses indicated that agreeableness and neuroticism were positively associated with fear of war. Individuals high in neuroticism are more likely to experience negative emotions such as fear, sadness, shame, anger, and guilt and generally report poorer psychological functioning (Anglim et al., 2020; Costa & McCrae, 2008). They also tend to rely more on passive and emotion-focused coping strategies (Lee-Baggley et al., 2005). Consistent with these characteristics, previous research has shown that neuroticism is associated with poorer coping during large-scale crises, including the COVID-19 pandemic (Burro et al., 2023), greater post-traumatic stress symptoms (Cohen-Louck & Zvi, 2022), and higher levels of anxiety and stress following adverse events (Di Crosta et al., 2020). Together, these findings suggest that individuals high in neuroticism may experience greater fear of war because they are more prone to negative affect and may have fewer psychological resources for coping with prolonged uncertainty and threat.

In terms of agreeableness, individuals high on this trait are typically described as compassionate, respectful, and attentive to others’ interests and tend to display cooperative and prosocial behavior in social interactions (Liu et al., 2025; Moore et al., 2022). Although the literature generally links high agreeableness to constructive conflict strategies, lower emotional reactivity, and higher subjective well-being (Anglim et al., 2020), the present findings point to a more context-dependent role of this trait. In this regard, the positive association between agreeableness and fear of war observed in this study may be explained by evidence suggesting that maintaining positive interpersonal relationships is a central motivational component of agreeableness (Jensen-Campbell & Graziano, 2001). Accordingly, one possible explanation is that individuals high in agreeableness may be more sensitive to the potential harm that war could cause to significant others, which may contribute to higher levels of fear of war. Although agreeable individuals often rely on constructive strategies such as compromise and negotiation in interpersonal conflict, such strategies may be less applicable in the context of uncontrollable and large-scale threats such as war, potentially increasing feelings of helplessness and fear. Consistent with this interpretation, Jensen-Campbell and Graziano (2001) suggested that individuals oriented toward group harmony may be more sensitive to interpersonal conflict. Similarly, a study conducted following the November 2015 Paris terrorist attacks reported a positive association between agreeableness and fear of terrorist attacks (Vasilopoulos & Brouard, 2020). In addition, individuals high in agreeableness have been shown to report higher levels of empathy and prosocial concern (Habashi et al., 2016), which may increase emotional sensitivity to the potential consequences of war for others. At the same time, alternative explanations should also be considered. For example, individuals high in agreeableness may be more willing to acknowledge and report fear-related emotions because of greater emotional awareness or differences in response style. Likewise, the observed association may reflect greater empathy and concern for others rather than greater personal fear of war itself. Because these possibilities were not directly examined in the present study, they remain tentative and warrant further investigation. Taken together, these findings suggest that the association between agreeableness and fear of war may be context-dependent and should be examined further across different types of collective threats.

Our analyses indicated that conscientiousness was significantly and negatively associated with fear of war. Individuals high in conscientiousness are typically careful, organized, efficient, and inclined to adhere to rules and recommendations, often adopting a more proactive approach when facing threat-related situations (Costa & McCrae, 2008; Fiddick et al., 2016; Bagherian & Mojambari, 2016). Conscientiousness has consistently been associated with problem-focused coping (Afshar et al., 2015), better adaptation during large-scale crises such as the COVID-19 pandemic (Burro et al., 2023; Moore et al., 2022), and higher subjective well-being (Anglim et al., 2020). Collectively, these findings are consistent with the possibility that conscientiousness may be associated with greater use of cognitive and behavioral resources that facilitate coping with war-related threats.

Our analyses indicated that extraversion and open-mindedness were not significantly associated with fear of war. Extraversion is generally characterized by sociability, assertiveness, and positive affect, whereas open-mindedness reflects curiosity, creativity, and openness to new experiences (McCrae & John, 1992; McAdams, 2015). Previous research has linked both traits to greater resilience and lower levels of post-traumatic stress symptoms (Cohen-Louck & Zvi, 2022), and open-mindedness has also been associated with better adaptation during the COVID-19 pandemic (Iterbeke & De Witte, 2022). However, these associations were not observed in the present study, suggesting that the influence of these personality traits on fear may depend on the specific characteristics of the threat and its broader context. This interpretation is consistent with evidence indicating that the effects of extraversion may vary across different stressful situations. For example, Kohút et al. (2021) reported that extraverted individuals experienced stronger negative emotional reactions during the COVID-19 pandemic, whereas Burro et al. (2023) found that extraversion was associated with both proactive coping and hopelessness. Similarly, Pineles et al. (2009) suggested that different facets of extraversion may have distinct associations with fear responses.

Similarly, although open-mindedness may facilitate individuals’ adaptation processes in the face of a threat, it may not constitute a core trait that directly determines fear responses in highly uncertain and threatening contexts such as war. Cohen-Louck and Zvi (2022) reported that open-mindedness is not directly associated with post-traumatic stress symptoms; however, it may exert an indirect protective effect through its positive association with ego resilience. The data support the view that the effects of personality traits on fear of war may be context-dependent. In line with Goldberg’s (1993) Five-Factor Model, personality traits represent relatively stable dispositions, whereas their emotional and behavioral expressions may vary depending on environmental conditions. Furthermore, Faulkner et al. (2004) demonstrated that environmental threats can shape individuals’ attitudes and responses, suggesting that the relationship between traits such as extraversion and open-mindedness and fear of war may be moderated by situational factors, including perceived threat level, exposure to information, and social context.

## 6. Practical Implications

Beyond establishing the psychometric properties of the Turkish version, the present study has several important practical implications. First, it provides clinicians, mental health practitioners, researchers, and public health professionals with a brief and reliable instrument for identifying individuals experiencing elevated fear of war, particularly in regions characterized by ongoing geopolitical instability or close proximity to armed conflicts. Early identification of elevated fear may facilitate timely psychoeducational and psychological interventions aimed at reducing subsequent psychological distress. Second, the scale may be useful in future longitudinal research examining changes in fear of war over time. However, further studies are needed to establish its responsiveness to change before its use for monitoring longitudinal change can be fully supported. In addition, the scale may assist preventive mental health initiatives targeting populations likely to experience heightened war-related stress, including university students, healthcare professionals, humanitarian workers, refugees, individuals with pre-existing mental health conditions, and communities living near conflict zones. Such early identification may support the development of targeted resilience-building, psychoeducation, and stress-management programs. Finally, the availability of a culturally validated instrument may contribute to mental health surveillance, preparedness planning, and evidence-informed intervention strategies in countries such as Türkiye, where geographical proximity to regional conflicts may increase public sensitivity to war-related threats.

## 7. Limitations

This study has several limitations that should be considered when interpreting the findings. First, both samples were recruited using convenience sampling, which may have introduced selection bias. In addition, the first sample predominantly comprised women, and both samples consisted primarily of young, unmarried adults. Consequently, the findings may not be fully generalizable to the broader Turkish population or to demographic groups that were underrepresented in the present study, including men, older adults, and married individuals. Second, because all variables were assessed using self-report questionnaires, the results may have been influenced by self-reporting biases, including social desirability, recall bias, and individual response tendencies. Moreover, collecting all measures from the same respondents at a single time point may have increased the risk of common method variance, potentially inflating the observed associations among the study variables. Third, although the translation procedure followed established forward–backward translation guidelines, formal qualitative evaluation of content validity (e.g., cognitive interviewing and assessments of comprehensibility, relevance, and comprehensiveness) was not conducted. Consequently, evidence regarding content validity remains incomplete. Future studies should strengthen this aspect of validation by incorporating qualitative evaluations involving members of the target population. Fourth, test–retest reliability was not examined; therefore, the temporal stability of the scale could not be evaluated. Consequently, absolute measurement error (e.g., the standard error of measurement [SEM], smallest detectable change [SDC], limits of agreement [LoA], and intraclass correlation coefficient [ICC]-based estimates) could not be evaluated. Future studies should assess temporal stability by administering the scale to the same participants across multiple time points and should also examine measurement error using these complementary indices. Finally, the cross-sectional design precludes causal inferences. Although personality traits and tolerance of uncontrollability were examined in relation to fear of war, the observed associations should be interpreted as correlational rather than causal. Future longitudinal studies using more diverse and representative samples together with multi-method assessment approaches are needed to clarify the temporal ordering and associations among these variables.

## 8. Conclusions

In the present study, we demonstrated that the Turkish version of the Fear of War Scale (FOWARS) possesses satisfactory psychometric properties and provided evidence for its structural validity, reliability, convergent validity, discriminant validity, criterion-related validity, and measurement invariance across gender. These findings support the scale’s use as a reliable instrument for assessing fear of war in Turkish populations. In addition, fear of war was found to be associated with psychological symptoms, tolerance of uncontrollability, selected personality traits, gender, and socioeconomic status. These findings contribute to a broader understanding of the psychological and demographic characteristics associated with fear of war, while highlighting the need for further longitudinal and multi-method research to better understand these associations. Given the persistence of armed conflicts in different parts of the world, the availability of a psychometrically sound Turkish version of the FOWARS may facilitate future research, psychological assessment, and the development of preventive mental health initiatives aimed at identifying and supporting individuals experiencing elevated fear of war.

## Figures and Tables

**Table 1:** Demographic characteristics of the participants.

Variable		First Sample		Second Sample	
		*N*	%	*n*	%
Gender	Female	297	82.5	158	55.8
	Male	63	17.5	125	44.2
Marital Status	Married	20	5.6	5	1.8
	Single	340	94.4	278	98.2
Socioeconomic Level	Low	59	16.4	46	16.3
	Middle	275	76.4	226	79.9
	Good	26	7.2	11	3.9

**Table 2:** Standardized factor loadings and average variance extracted (AVE) for the FOWARS.

Factor	Indicator	Std. Estimate	Std. Error	z-Value	*p*	Mean	SD
Experiential (AVE = 0.572) Composite reliability (CR = 0.90)	f1	0.765	0.044	17.29	<0.001	3.85	1.102
	f2	0.835	0.047	17.79	<0.001	3.84	1.084
	f3	0.770	0.043	18.10	<0.001	3.62	1.138
	f4	0.722	0.044	16.29	<0.001	3.93	1.106
	f5	0.770	0.043	17.79	<0.001	3.77	1.159
	f6	0.677	0.046	14.68	<0.001	4.07	1.006
	f7	0.732	0.040	18.17	<0.001	3.78	1.123
Physiological (AVE = 0.666) Composite reliability (CR = 0.92)	f8	0.846	0.035	23.88	<0.001	2.63	1.256
	f9	0.836	0.035	23.74	<0.001	2.48	1.268
	f10	0.914	0.037	24.61	<0.001	2.67	1.256
	f11	0.697	0.035	20.00	<0.001	2.22	1.176
	f12	0.830	0.036	23.34	<0.001	2.85	1.231
	f13	0.739	0.036	20.73	<0.001	2.25	1.192

**Table 3:** The relationships between fear of war and tolerance of uncontrollability, depression, anxiety, and stress.

Variable	1	2	3	4	5
Fear of War	1				
Tolerance of Uncontrollability	−0.21 ***	1			
Depression	0.21 ***	−0.08	1		
Anxiety	0.29 ***	−0.12 *	0.75 ***	1	
Stress	0.24 ***	−0.13 *	0.71 ***	0.73 ***	1
Mean	43.23	70.08	7.48	7.06	8.36
SD	10.48	22.60	4.57	4.70	4.23
Skewness	−0.17	0.15	0.45	0.58	0.50
Kurtosis	0.01	−0.30	−0.17	0.16	0.06

Note: *** *p* < 0.001; * *p* < 0.05.

**Table 4:** Measurement invariance of the FOWARS across gender.

Models	χ^2^ (df)	χ^2^/df	CFI	SRMR	RMSEA	Model Comparison			
							∆χ^2^ (∆df)	∆CFI	∆RMSEA
1. Configural	282.520 (128)	2.207	0.948	0.075	0.082				
2. Metric	298.734 (139)	2.149	0.942	0.081	0.080	2 vs. 1	16.214 (11)	<0.01	<0.015
3. Scalar	355.143 (150)	2.368	0.934	0.079	0.087	3 vs. 2	56.409 (11)	<0.01	<0.015

**Table 5:** Hierarchical multiple linear regression examining associations between sociodemographic characteristics, personality traits, and fear of war.

Variable	Model I					Model II				
	B	SE	*β*	t	*p*	B	SE	*β*	t	*p*
Constant	35.13	9.59		3.66	<0.001	27.72	11.95		2.32	0.021
Sociodemographic characteristics										
Age	0.06	0.24	0.02	0.26	0.796	0.11	0.24	0.03	0.48	0.630
Gender	5.87	1.50	0.24 ***	3.92	<0.001	4.45	1.45	0.18 **	3.07	0.002
Socioeconomic level	5.84	1.74	0.21 ***	3.36	<0.001	3.81	1.73	0.14 *	2.21	0.028
Marital status	−2.74	5.89	−0.03	−0.47	0.642	−5.79	5.72	−0.06	−1.01	0.313
Big Five personality traits										
Extraversion						0.10	0.31	0.02	0.31	0.761
Agreeableness						1.53	0.35	0.26 ***	4.40	<0.001
Conscientiousness						−0.74	0.31	−0.17 *	−2.41	0.017
Neuroticism						0.65	0.33	0.13 *	2.01	0.046
Open-mindedness						−0.45	0.33	−0.10	−1.38	0.168
*R* ^2^	0.146					0.245				
Adjusted *R*^2^	0.133					0.221				
F for Change in *R*^2^	11.859 ***					7.211 ***				

Note: * *p* < 0.05; ** *p* < 0.01; *** *p* < 0.001.

## Data Availability

The data supporting the findings of this study are available from the corresponding author upon reasonable request.
